# Enhanced Intelligent Identification of Concrete Cracks Using Multi-Layered Image Preprocessing-Aided Convolutional Neural Networks

**DOI:** 10.3390/s20072021

**Published:** 2020-04-03

**Authors:** Ronghua Fu, Hao Xu, Zijian Wang, Lei Shen, Maosen Cao, Tongwei Liu, Drahomír Novák

**Affiliations:** 1 Department of Engineering Mechanics, Hohai University, Nanjing 210098, China; ronghua@hhu.edu.cn (R.F.); leishen@hhu.edu.cn (L.S.); twliu@hhu.edu.cn (T.L.); 2 School of Aeronautics and Astronautics, Faculty of Vehicle Engineering and Mechanics, State Key Laboratory of Structural Analysis for Industrial Equipment, Dalian University of Technology, Dalian 116024, China; xuhao@dlut.edu.cn; 3 Department of Civil and Environmental Engineering, Northwestern University, Chicago, IL 60626, USA; 4 Key Laboratory of C&PC Structures, Ministry of Education, Southeast University, Nanjing 211189, China; wzj@seu.edu.cn; 5 Jiangxi Provincial Key Laboratory of Environmental Geotechnical Engineering and Disaster Control, Jiangxi University of Science and Technology, Ganzhou 341000, China; 6 Institute of Structural Mechanics, Faculty of Civil Engineering, Brno University of Technology, 60200 Brno, Czech Republic; novak.d@fce.vutbr.cz

**Keywords:** concrete crack identification, convolutional neural network, homomorphic filtering, structural health monitoring, signal processing

## Abstract

Crack identification plays an essential role in the health diagnosis of various concrete structures. Among different intelligent algorithms, the convolutional neural networks (CNNs) has been demonstrated as a promising tool capable of efficiently identifying the existence and evolution of concrete cracks by adaptively recognizing crack features from a large amount of concrete surface images. However, the accuracy as well as the versatility of conventional CNNs in crack identification is largely limited, due to the influence of noise contained in the background of the concrete surface images. The noise originates from highly diverse sources, such as light spots, blurs, surface roughness/wear/stains. With the aim of enhancing the accuracy, noise immunity, and versatility of CNN-based crack identification methods, a framework of enhanced intelligent identification of concrete cracks is established in this study, based on a hybrid utilization of conventional CNNs with a multi-layered image preprocessing strategy (MLP), of which the key components are homomorphic filtering and the Otsu thresholding method. Relying on the comparison and fine-tuning of classic CNN structures, networks for detection of crack position and identification of crack type are built, trained, and tested, based on a dataset composed of a large number of concrete crack images. The effectiveness and efficiency of the proposed framework involving the MLP and the CNN in crack identification are examined by comparative studies, with and without the implementation of the MLP strategy. Crack identification accuracy subject to different sources and levels of noise influence is investigated.

## 1. Introduction

Concrete structures in various forms, such as high-rise buildings, bridges and dams, suffer from continuous health deterioration during long service periods [1,2], making real-time detection of different types of structural damage a crucial demand [3]. Traditional visual inspection methods relying on human labor for damage diagnosis entail unavoidable limitations such as high dependence on individual subjectivity, expertise, and an extensive amount of labor [4]. In past decades, image processing techniques demonstrated great advantages in aspects of efficiency, accuracy, objectivity [5], etc., and were widely adopted to extract damage features, where crack recognition from concrete surface images formed a particularly active area. Some representative methods for crack identification include the threshold segmentation method, edge detection method, and artificial intelligence (AI) method. A threshold segmentation algorithm proposed by Otsu [6] has been widely used in various image recognition tasks [7], and modifications of that algorithm have been made for crack identification [8,9,10,11,12]. For instance, considering noise influence as the major obstacle for accurate image recognition, Migdal et al. [13] conducted noise suppression with the assistance of Markov random fields of binary segmentation, taking into account neighboring pixel classifications. While the process produced a prominent noise reduction effect, the features of crack and noise become difficult to distinguish with an increase in the extent of noise interference. Image edge detection was also adopted for crack recognition, with representative applications established on Roberts, Prewitt and Sobel operators [14]. However, the edge detection algorithm caused a typical ill-posed problem in that it was highly sensitive to noise influence, especially due to light and distortion, and optimal solutions were difficult to obtain [15]. Modifications have been made to address the above issues. For example, noise removal algorithms of nonlinear total variation proved effective in noise reduction and thus were able to enhance the accuracy of edge detection [16]; Cha et al. [17] used Canny edge detection with a classifier of linear support vector machine to distinguish loosened bolts from intact ones; Ayenu-Prah et al. [18] employed the Sobel edge detection algorithm using bidimensional empirical mode decomposition for crack identification and noise suppression; Acharya et al. [19] reduced noise using gradient-based edge detection based on the difference between edge and non-edge pixels, where images in a color filter array were treated; Yan et al. [20] developed an edge detection algorithm based on morphological filtering set to overcome the interference of noise; Zhou et al. [21] adopted an improved edge detection algorithm based on a wavelet transform to detect pavement distress, showing an advantage in quantifying the degree of damage. However, the accuracy of the aforementioned algorithms is still considered limited in crack identification when taking into account severe noise influences that are common in actual damage detection scenarios.

With rapid development in recent years, AI methods have been increasingly applied in structural damage detection [22], where crack recognition based on convolutional neural networks (CNNs) has shown promise in engineering applications [23]. Compared to conventional machine learning methods, CNNs are particularly powerful in learning the characteristics of images using a simpler network structure [24]. Leveraging this merit, CNN-based methods can identify cracks with high efficiency, especially when dealing with multi-classification [25] and large-scale problems [26]. Moreover, relying on transfer learning, existing CNN structures can easily be modified and well utilized to solve similar types of problems [27], enhancing the adaptability of CNNs for treating different crack images. Chen et al. [28] proposed a method based on CNNs and naïve Bayes data fusion to detect cracks in a single frame of video; Browne et al. [29] suggested the implementation of separable filters to detect sewer cracks based on a CNN model. More applications can be found in the studies of Cha [30], Zhao [31], Zhang [32], and Gavilán et al. [33], where the key concepts of crack detection were analogous. That is, the image was first divided into a number of sub-regions, from which crack features were extracted to form the feature vectors. The CNN was trained based on these feature vectors and then used to identify cracks in each sub-region of the full image. Finally, the crack in the full image was recognized by combining the identification results of each sub-region. While possessing inherent noise immunity, CNNs still face challenges in accurate identification of concrete cracks [34]. That is because the image background noise exhibits a large number of distinct features, which increase the difficulty of crack feature extraction and largely jeopardize the accuracy, efficiency, and versatility of CNNs. Unfortunately, as commonly encountered in actual practice, severe background noise originating in concrete surfaces from complex and diverse sources can hardly be avoided.

In this paper, crack identification from images of concrete surface was carried out based on the hybrid utilization of CNNs and a multi-layered image preprocessing (MLP) strategy, defined as the MLP–CNN framework, the central components of which are homomorphic filtering and the Otsu thresholding method. Apart from clear improvement in detection accuracy and noise immunity, the MLP–CNN framework is also able to strengthen the versatility of crack identification, meaning that different types and levels of background noise can be treated using a uniform framework. Specifically, both crack position detection (CPD) and crack type identification (CTI) networks were built, trained and tested, based on datasets composed of a number of concrete crack images. The effectiveness and efficiency of the developed framework was examined by comparing crack detection results with and without the implementation of the MLP strategy. Crack identification accuracy subject to different sources and levels of noise influence was investigated. 

## 2. MLP–CNN Framework

**Figure 1:**
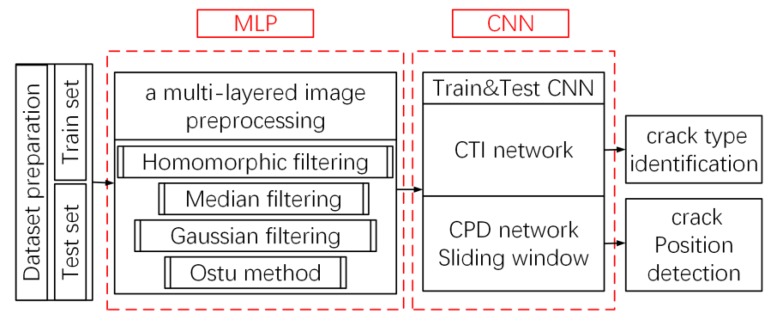
Diagram of the overall architecture of the MLP–CNN framework.

**Figure 2:**
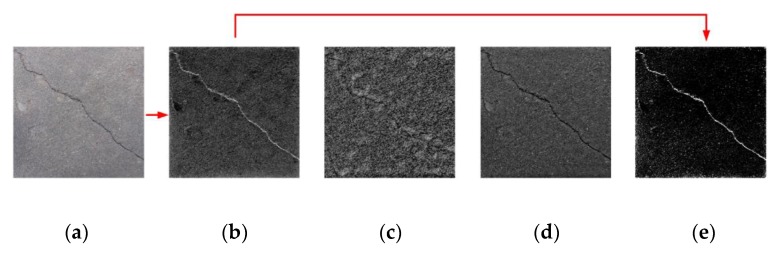
(**a**) Original image containing a crack, and the crack feature extraction results treated by: (**b**) Gaussian filter; (**c**) Butterworth filter; (**d**) ideal high pass filter for homomorphic filtering; and (**e**) the Otsu thresholding method.

The architecture of the proposed MLP–CNN framework is shown in Figure 1. The multi-layered preprocessing structure includes a combination of filtering and feature extraction techniques applied in sequence to fulfill different functions. The first layer, one of the key components in the MLP, is homomorphic filtering [35], used to process concrete surface images in the frequency domain. For image processing, homomorphic filtering is able to suppress low frequency components, such as those associated with lighting variations, while highlighting high-frequency components associated with local details such as crack edges. Considering that frequency-domain filtering can cause oscillation of the grayscale in the output image, known as the ringing effect [36], the selection of filter types in homomorphic filtering has a direct impact on the denoising effect. Three high-pass filters commonly used for homomorphic filtering are the Gaussian filter, Butterworth filter, and ideal high pass filter. As can be seen in Figure 2b, Gaussian filtering produces the optimal denoising effect, by showing the significant crack feature with minimal ringing effect. In Figure 2c,d, the crack features are largely submerged in the backgrounds. Figure 2c, treated by the Butterworth filter, is severely contaminated by high-frequency noise likely associated with the ringing effect; Figure 2d, treated by the ideal high pass filter, shows too weak contrast in the colors of the crack and background. These unsatisfactory results are due to the filter characteristics, such as the dramatic changes of gradient in the filter functions. Thus, the Gaussian filter was adopted in this study for homomorphic filtering.

The other key layer of the MLP structure is the Otsu thresholding algorithm [6] used to identify the maximum value of gray in the image. Image binarization is carried out in accordance with the adaptive selection of thresholds. As can be seen in Figure 2e, the Otsu thresholding method, which is applied after the homomorphic filtering, transforms the crack images into binarized images with prominent crack features highlighted.

The causes of background noise in the images can be complex, due to unexpected situations. Moreover, additional noise may be generated during the filtering process to distort the image. Thus, to obtain the optimal de-noising effect, two additional layers, median filtering and a Gaussian filter, were inserted between the homomorphic filtering layer and the Otsu thresholding layer. Median filtering is a nonlinear signal-processing technique based on the theory of sorting statistics. With good noise suppression effect, median filtering is particularly effective in maintaining crack edge information, due to its demonstrated merit in image edge protection [37]. The Gaussian filter, with the smooth feature of the Gaussian function, is applied next to process the image, reducing the accumulation of errors during filtering. By using the MLP structure, concrete surface images containing different noise types and levels can be rapidly transformed into binary images with significant crack features, and thus the generation abilities of the CNNs applied subsequently are maximized.

In the following study, the construction and application of the CNNs are introduced in Section 3, where CPD and CTI networks were built to identify the position and type of cracks, respectively. The constructions of these two CNNs are conducted in a similar way to that of the CNN-based methods introduced in the literature. In Section 4, comparative studies are presented, showing crack identification results achieved with and without the implementation of the MLP.

## 3. Concrete Crack Identification Based on CNN

### 3.1. Overall Structure

**Figure 3:**
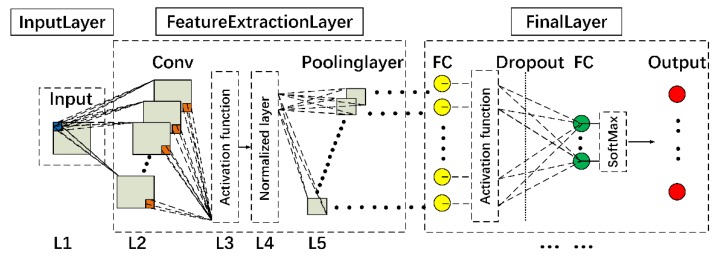
Overall structure of CNN.

A general CNN structure is shown in Figure 3. The structure is mainly composed of three layers, the input layer, the feature extraction layer and the final layer, where the sub-layers in the feature extraction layer (i.e., L2, L3, L4, and L5) can be repeated by considering practical demands. For specific crack detection aims, two network types with analogous structures were constructed, defined as crack position detection (CPD) and crack type identification (CTI) networks, respectively.

#### 3.1.1. Feature Extraction Layer

**Figure 4:**
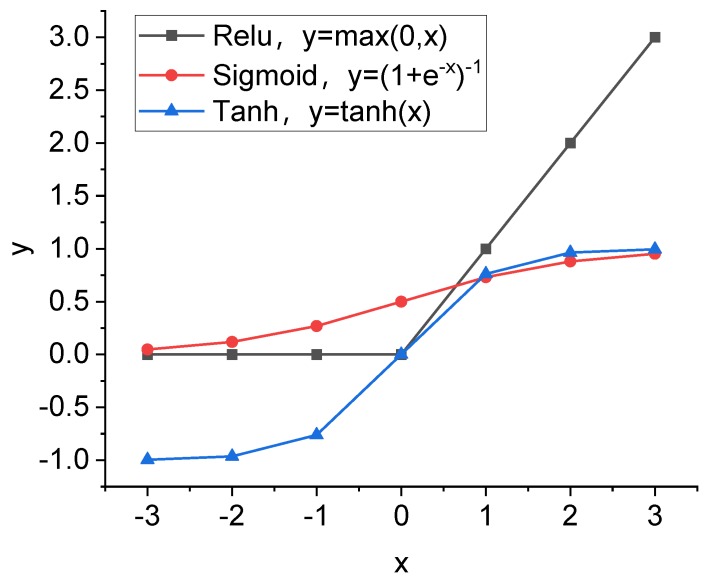
Examples of commonly used activation functions for neural networks.

The feature extraction layer consists of multiple convolutional layers (CONVs) and pooling layers. The CONVs (in L2 as shown in Figure 3) feature sparse connections between the convolution kernels and the pixel matrices of the input images, enabling efficient network training that benefits from the relatively small number of network parameters [38]. Besides, the memory usage for computation can be reduced due to the weight sharing of the CONVs [39]. Initial weights and bias of the convolution kernel in L2 were generated randomly. Activation functions (L3) were used to introduce nonlinear characteristics into the network [40]. Examples of nonlinear functions are shown in Figure 4. As can be seen, the sigmoid function ranging between 0 and 1 shows a small gradient that can result in a slow convergence speed and the problem of the gradient vanishing during CNN training. The hyperbolic tangent (tanh) ranging between -1 and 1 shows a larger gradient than that of the sigmoid and thus is easier for optimization. The rectified linear units (ReLU), in the simple mathematical form as shown in Figure 4, has the largest gradient, which can increase the training efficiency and accuracy of CNN [41]. Therefore, ReLU was selected as the activation function in the following study, located after the CONVs shown as L3 in Figure 3.

A normalized layer (shown as L4 in Figure 3) is often included in CNN to enhance the generalization ability of the network, by creating a competitive mechanism for local neuron activity [42]. The present crack recognition network adopted batch normalization (BN) (the advantage of which was demonstrated in [43]) as the normalized layer to alleviate the gradient vanishing problem in network training. The pooling layer, shown as L5 in Figure 3, was constructed to perform aggregate statistics, based on the location features of different images, to further reduce data complexity as well as the probability of overfitting. The MaxPooling method was adopted for crack recognition.

#### 3.1.2. Final Layer

The final layer normally contains multiple fully connected layers (FCs), activation functions and a softmax layer. Dropout layers may also be included for improvement of accuracy. Dropout reduces co-dependence between nodes by randomly resetting parts of the weights or outputs of the fully connected layer to zero during the CNN training process, whereby the problem of overfitting can be prevented [44]. As can be seen in Figure 3, the Dropout layer is often between FCs in the final layer.

The output of the softmax layer indicates the probability of an object being classified into a category. The softmax layer is located before the output layer and is usually adopted for multi-classification problems (i.e., C > 2). The final output of the network requires utilization of a softmax function to generate the categories of objects with the highest probability. The softmax function is defined as:

(1) $$S_{i}=\frac{e^{V_{i}}}{\sum_{i}^{C}e^{V_{i}}}.$$

where *S_i_* is the softmax function; *Vi* is the *i*^th^ element in the set *V* to be classified; *C* is the total number of categories. Softmax is often located at the end of final layer and is connected to the output layer, representing the relative probability of different categories [45]. 

#### 3.1.3. Training Algorithms

During the training process, forward propagation obtained a loss, as the differences between the output and the real values, by evaluating which of the CNN parameters were optimized. Specifically, a stochastic gradient descent was used to minimize the cost function [46] and achieve optimal biases and weights. The cost function of the *i*^th^ CNN layer is:

(2) $$J^{\left(i\right)}\left(\theta_{0},\theta_{1}\right)=\frac{1}{2}{\left(h_{\theta }\left(x^{\left(i\right)}\right)-y^{\left(i\right)}\right)}^{2},$$

the partial derivative of which is:

(3) $$\frac{∆J^{\left(i\right)}\left(\theta_{0},\theta_{1}\right)}{\theta_{j}}=\left(h_{\theta }\left(x^{\left(i\right)}\right)-y^{\left(i\right)}\right)x_{j}^{\left(i\right)},$$

and the parameter updating formula is written as:

(4) $$\theta_{j}≔\theta_{j}-\alpha \frac{∆J^{\left(i\right)}\left(\theta_{0},\theta_{1}\right)}{\theta_{j}}.$$

In Equations (2)–(4), *i* represents the CNN neuron index; *j* represents the CNN layer index and *α* is the learning rate. Note that each iteration of the gradient descent algorithm is affected by *α*. $\theta_{0}$ and $\theta_{1}$ are the weight and bias of the CNN neurons to be updated; $h_{\theta }\left(x^{\left(i\right)}\right)$ is the predicted value of the CNN output, and $y^{\left(i\right)}$ is the true value.

### 3.2. Construction of the CPD Network

#### 3.2.1. Preparation of Data Sets

**Figure 5:**
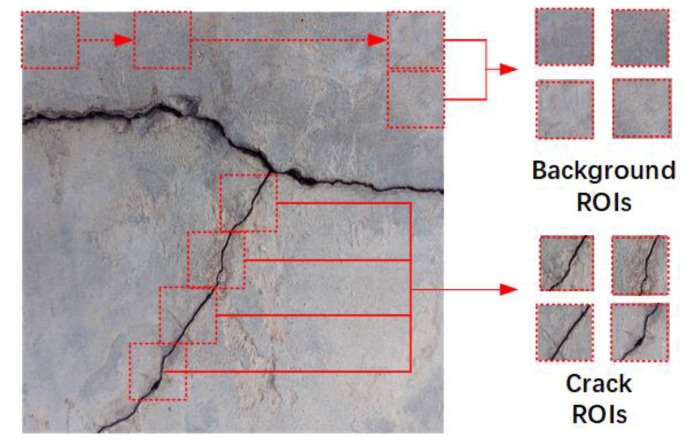
Diagram illustrating the method for preparing datasets for the CPD network, which consists of crack and background regions of interest (ROIs).

The images for crack detection were live pictures of different concrete surfaces, each containing 1024 × 1024 pixels. The full-size images were divided into small sub-regions, defined as regions of interest (ROIs), with the uniform resolution of 32 × 32. ROIs containing cracks are called crack ROIs and all the others are called background ROIs, as shown in Figure 5. The training set for the CPD network included 1500 crack ROIs and 5000 background ROIs, whereas the test set included 542 crack ROIs and 3680 background ROIs.

#### 3.2.2. Structure of the CPD Network

**Figure 6:**
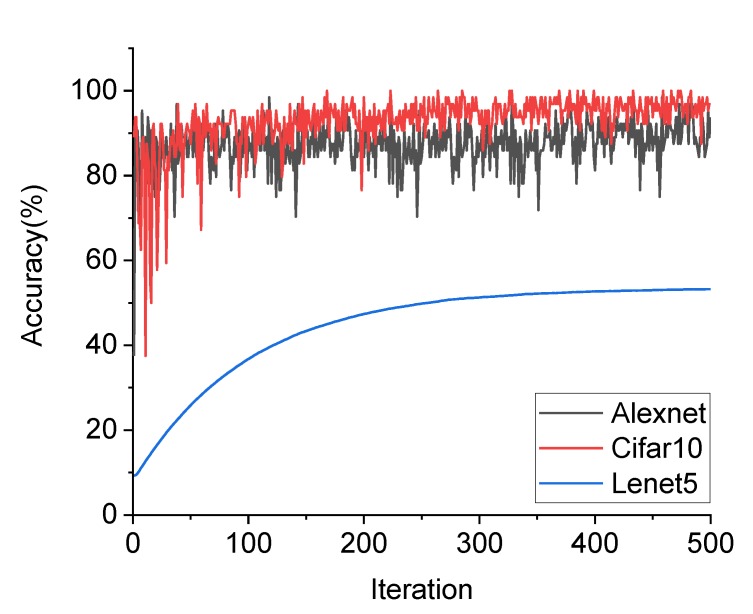
Accuracy variations during the training process using Alexnet, Lenet5, and CIFAR10, respectively.

Because the CPD network was designed to deal with two-classification problems, a simple network structure was preferred to meet the requirement of training efficiency without an overfitting problem. Three classical CNN structures for two-dimensional image recognition, Alexnet, Lenet5 and CIFAR10 [47], were tested for accuracy of identification of ROIs along with the training process, as shown in Figure 6. Table 1 presents the ultimate accuracy and total time of training. From observation of Figure 6 and Table 1, Lenet5 shows the lowest recognition accuracy for crack and background identification, whereas Alexnet shows the highest accuracy. However, the training of Alexnet is very time-consuming because of its complex structure. Therefore, the CIFAR10 network can be considered a favorable option for establishing a CPD network, due to its balance of efficiency and accuracy. More importantly, with its simple structure, the CIFAR10 network can be easily adjusted based on finely tuned parameters to achieve improvement of accuracy. Nevertheless, Alexnet was used to construct the CTI network, as will be introduced. 

**Figure 7:**
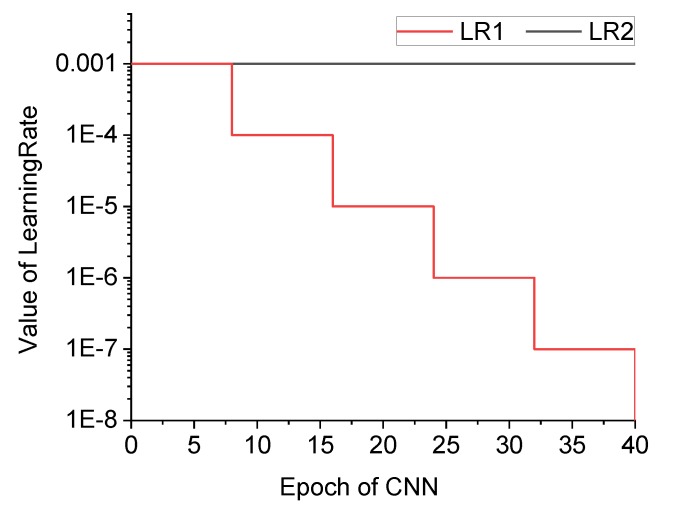
Two learning rate curves during the network training period.

Three adjusted structures based on CIFAR10, referred to as Cracknet1-1, 1-2 and 1-3, were tested for comparison. Cracknet1-1 has a feature extraction layer including three CONVs, three pooling layers, and three ReLU activation functions, without normalization and dropout layers. The learning rate (i.e., α in Equation (4)) of Cracknet1-1 is marked as LR1 in Figure 7, with the initial value of 0.001, which is multiplied by a scale factor of 0.1 every 8 epochs. Cracknets1-2 and 1-3 are similar to 1-1, except that the former includes a dropout layer, whereas the latter adopts the constant learning rate of LR2 (=0.001) as shown in Figure 7. The ultimate accuracy of Cracknets1-1, 1-2, and 1-3 were 94.6%, 95.8% and 94.4%, respectively. It can be seen that, with the addition of a dropout layer, the accuracy of Cracknet1-2 increased about 1% compared with that of Cracknet1-1. On the other hand, the learning rate variation had little impact on the accuracy of the CPD network. However, CNN is sometimes difficult to converge, which requires the learning rate to decrease along with the training epoch to achieve better convergence. Thus, LR1 was the preferred option in this study. Therefore, by considering the overall performance in both accuracy and learning rate, Cracknet1-2 was selected as the CPD network, with detailed information as shown in Table 2. Apart from the extraction layer as already introduced, the final layer consisted of two FCs, one dropout layer, one ReLU activation function and one softmax layer connected to the output layer. There were 40 epochs, with 96 iterations per epoch, for the training of the network.

### 3.3. Crack Position Detection Using Sliding Window

**Figure 8:**
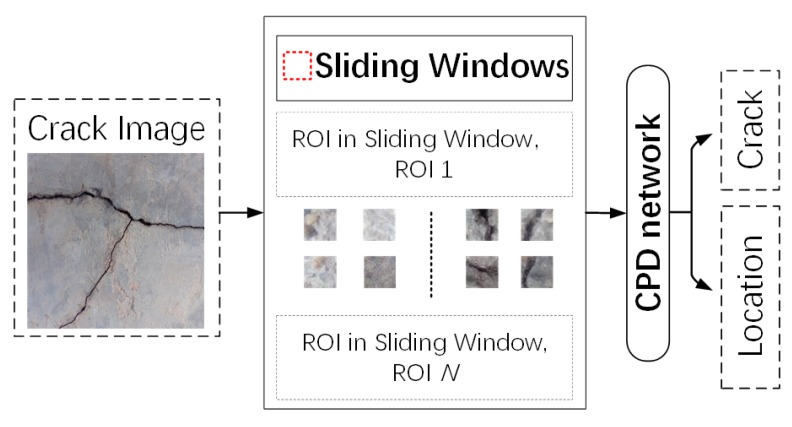
Schematic diagram of the procedure of crack position detection using a sliding window.

To detect cracks using the CPD network, the image was scanned by a sliding window as shown in Figure 8. The window size was 32 × 32 which is the same as that of the ROIs. With ROI types (i.e., crack or background) and coordinates as the outputs, crack positions were identified in terms of the coordinates of the crack ROIs.

**Figure 9:**
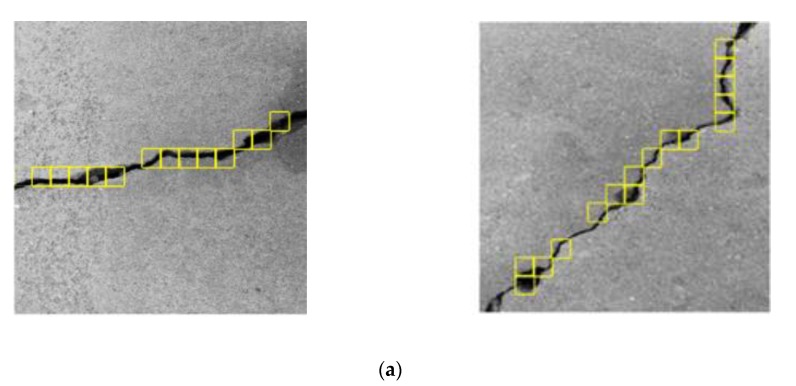

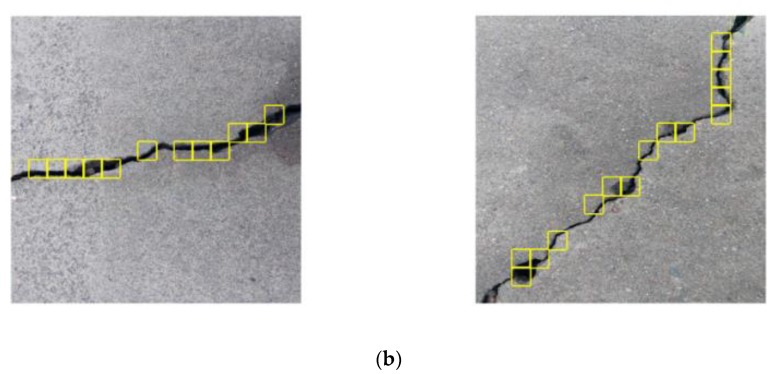
Crack position detection (CPD) results using the (**a**) constructed CPD and (**b**) Alexnet network.

Figure 9 shows the crack position detection results using the CPD network or the Alexnet classifier. The sliding step of the window was set at 32 pixels, which means that no overlap exists between two adjacent windows. It can be seen that the constructed CPD network achieves detection accuracy similar to that of the Alexnet, despite using a simpler structure.

**Figure 10:**
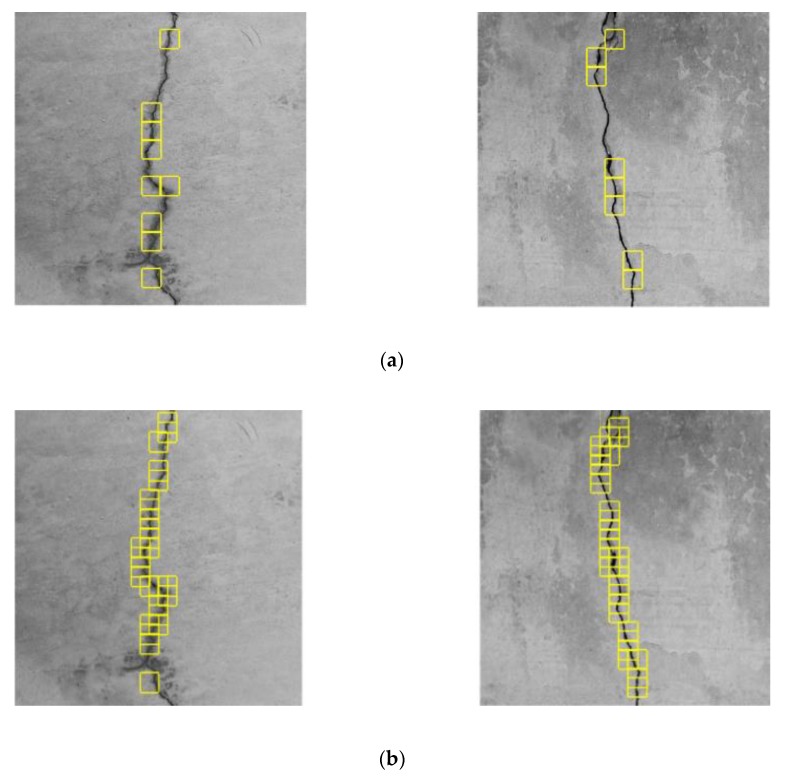
Crack position detection results subject to (**a**) 32 and (**b**) 16 pixels in step size of the sliding window.

The impact of variation of the sliding step on crack recognition accuracy is shown in Figure 10, where a different set of surface images is used. It can be seen that, with the sliding step of 32 pixels, the crack recognition results exhibit several discontinuities, losing important information in the main portions of the cracks. With reduction of the step size to 16 pixels, on the other hand, recognition accuracy is considerably enhanced. This is because, with reduction of the step size, adjacent windows begin to overlap to contain some same areas of interest [48], which are repeatedly identified by the CPD network to increase the possibility of correct crack recognition.

### 3.4. Construction of the CTI Network

#### 3.4.1. Preparation of Data Sets

**Figure 11:**
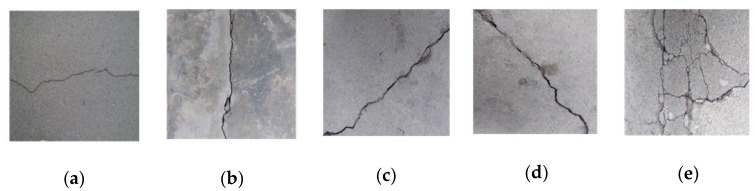
Five crack types: (**a**) transverse cracks; (**b**) vertical cracks; (**c**) left oblique cracks; (**d**) right oblique cracks; (**e**) mesh cracks.

Unlike the data sets of the CPD network, consisting of crack and background ROIs, the data sets of the CTI network consisted directly of full images of the concrete surface. To improve training efficiency, the resolution of the original images was reduced from 1024 × 1024 to 227 × 227. Five crack types, transverse cracks, vertical cracks, left oblique cracks, right oblique cracks, and mesh cracks, were defined, with examples shown in Figure 11. Therefore, the CTI was constructed as a five-classification network without the assistance of a sliding window. The training and test sets, which were under moderate noise influence, contained 1500 and 577 images, respectively. 

#### 3.4.2. Structure of CTI Network

**Figure 12:**
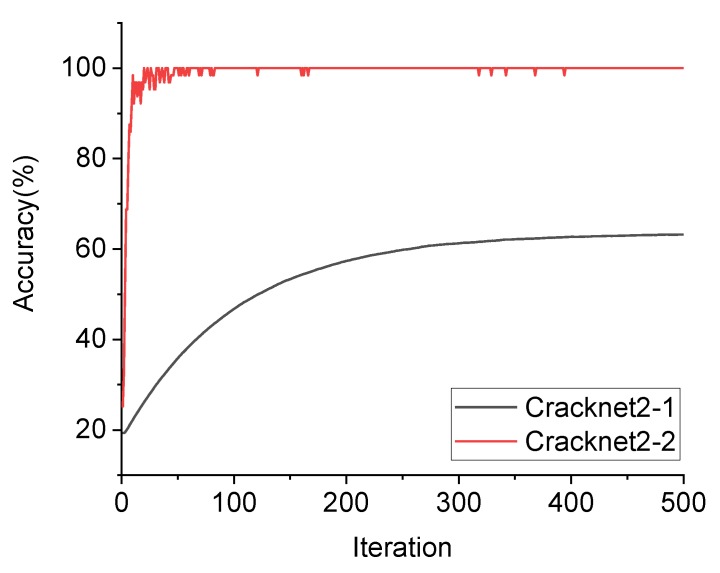
Accuracy variations along the training process using Cracknet2-1 and Cracknet2-2, respectively.

Two structures, labeled Cracknet2-1 and Cracknet2-2, were constructed and compared. Cracknet2-1 was based on Lenet5, with the feature extraction layer consisting of two CONVs and two pooling layers. Cracknet2-2 was based on Alexnet structure, with the learning rate LR1 selected, containing 40 epochs including 13 iterations per epoch during the training of Cracknet2-2, as shown in Figure 7. The structure of Cracknet2-2 is shown in Table 3. The feature extraction layer includes five CONVs, three pooling layers, five ReLU activation functions, and two batch normalization layers. The final layer consists of three FCs, two ReLU activation functions, two dropout layers, and a softmax layer connected to the output layer. Figure 12 compares the accuracy of Cracknets 2-1 and 2-2 for crack type identification during the training process. The ultimate accuracy levels of Cracknets 2-1 and 2-2 are 34.2% and 99.3%, respectively. The superiority of Cracknet2-2 can easily be seen.

For comparison, Cracknet2-2 was further tuned to obtain another two structures, Cracknets 2-3 and 2-4, in which 2-3 used a different learning rate and 2-4 deleted the dropout layer. The ultimate accuracy levels of Cracknets 2-3 and 2-4 were 98.4% and 98.9%, respectively. It can be seen that under the learning rate of LR1 and the dropout layer, Cracknet2-2 still gave the best results, and was thus selected as the CTI network. 

**Figure 13:**
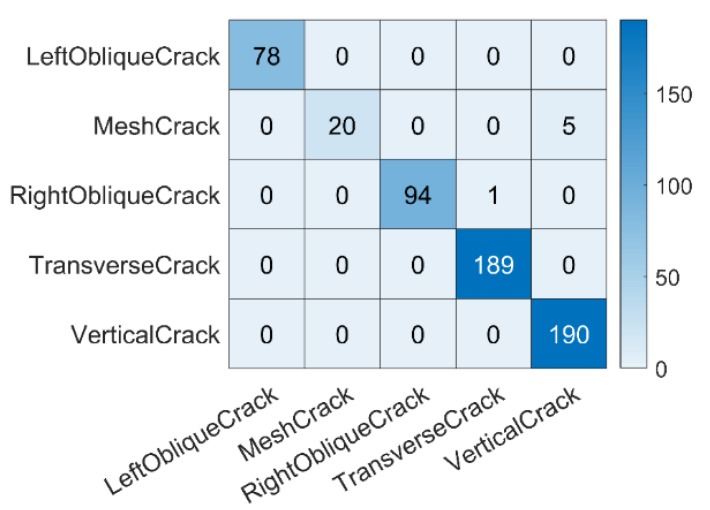
Confusion matrix constructed based on the crack type identification results based on the CTI network.

To examine the effectiveness of crack recognition by the CTI network (i.e., Cracknet 2-2), a confusion matrix [49] that is often used to evaluate the quality of classifiers was constructed to quantify the classification result, as shown in Figure 13. The horizontal and vertical elements of the matrix are the true and predicted values, respectively. The sum of each row represents the actual number of samples in a given class, whereas the sum of each column represents the number of samples predicted by the network. According to the matrix, the accuracy of classification can be deemed high, although false classification does occur in certain cases. That is, 20 (out of 25) cases of mesh crack were correctly identified, but five cases were wrongly recognized as vertical cracks; 94 (out of 95) cases of right oblique cracks were correctly identified, with one case wrongly recognized as a transverse crack. The overall accuracy of the CTI network was estimated to be 99.3%.

**Figure 14:**
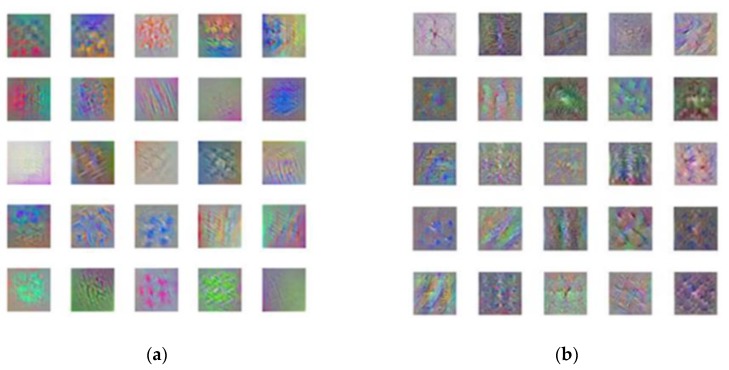
Visualization of crack features in the (**a**) second and (**b**) third convolutional layer.

More detailed analysis was conducted to examine the first 25 feature maps of the 2nd and 3rd convolutional layers in the CTI network, as shown in Figure 14. Based on convolution visualization, the crack features extracted by convolution kernels can be seen, showing information about edge, direction, textures, color, etc. Although each type of crack has similar color patterns, the patterns of crack edges and textures show larger differences, and difference in the direction patterns is significant. For the given classification problem, prominent crack direction patterns give rise to the high recognition accuracy of 99.3% using the CTI network.

## 4. Concrete Crack Recognition Based on the MLP–CNN Framework

Based on the establishment of the CTI and CPD networks and the sliding window strategy for crack detection, as introduced in Section 3, the MLP–CNN framework was used in this section, focusing on processing different types and levels of the influence of image noise.

### 4.1. Comparison among Feature Extraction Algorithms

The background noise in concrete surface images can exhibit complex and diverse forms that greatly increase the difficulty of crack recognition. For example, photography of cracked concrete surface is easily interfered with by factors such as a light spot, or a blur caused by camera shake (where an extreme case is drone photography of dams, bridges, roads, etc.). Other factors, such as rain erosion, daily wear, or human interference can lead to largely uneven concrete surfaces or stains. It was shown in Section 3 that the effectiveness of the CPD and CTI network was satisfactory under relatively low levels of background noise. However, severe noise results in a large decrease in the crack detection accuracy of both traditional image feature extraction methods and CNN-based methods.

**Figure 15:**
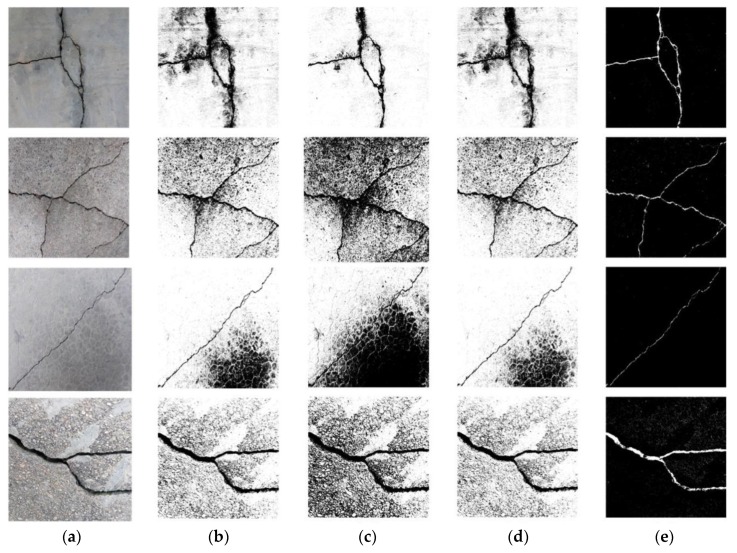
Comparison of crack feature extraction algorithms, subject to (**a**) no treatment; (**b**) linear enhancement filtering method; (**c**) iterative threshold segmentation method; (**d**) bit plane slicing; (**e**) MLP proposed in this paper.

Figure 15 shows the results of crack feature extraction obtained by different preprocessing techniques, i.e., the linear enhancement filtering method, the iterative threshold segmentation method, the bit plane slicing method, and the MLP used in this study. Subject to relatively severe background noise, the apparent drawbacks of conventional crack extraction algorithms can be seen in the figure, where it is difficult to effectively differentiate the features of noise from those of the cracks. Moreover, there are considerable differences between the feature extraction capacities of the different methods. The results of the MLP, on the other hand, reveal significant crack features by minimizing the noise interference. More importantly, with its binary nature and high precision, the MLP shows considerable versatility in treating different noise types and levels and thus can be combined with CNN to detect cracks under different circumstances.

**Figure 16:**
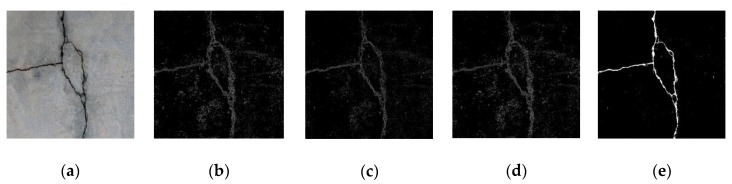
Comparison between edge detection and MLP, subject to (**a**) no treatment; (**b**) Prewitt operator; (**c**) Roberts operator; (**d**) Sobel operator and (**e**) MLP proposed in this study.

Comparison results between the MLP-based results and the results of edge detection algorithms (based on Prewitt, Roberts and Sobel operators, respectively) are shown in Figure 16. It can be observed that the MLP-treated results are able to reveal much more prominent crack features.

### 4.2. Crack Position Detection Subject to Moderate Noise Level

**Figure 17:**
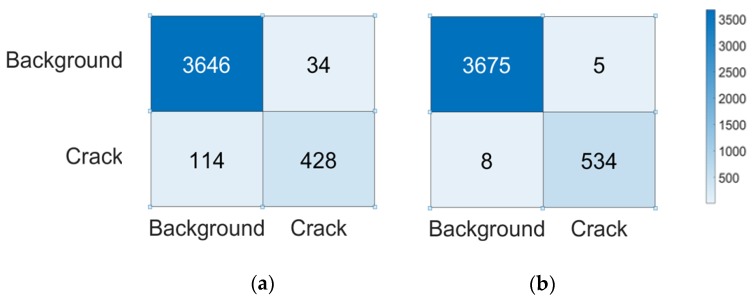
Confusion matrix constructed based on the recognition results using (**a**) the original CPD and (**b**) the MLP–CNN.

It was found that, with use of the MLP–CNN framework, the accuracy of the CPD network increased from 96.5% to 99.6%. A straightforward illustration is shown in Figure 17, where the confusion matrix in Figure 17a shows that the original CPD network results in 114 (out of 542) crack ROIs that are misclassified as background ROIs, and of the background ROIs, 34 (out of 3680) are misclassified as crack ROIs. As shown in Figure 17b, with the use of the MLP–CNN, instances of misclassification are largely reduced, i.e., only 8 (out of 542) crack ROIs and 5 background ROIs are misclassified. 

**Figure 18:**
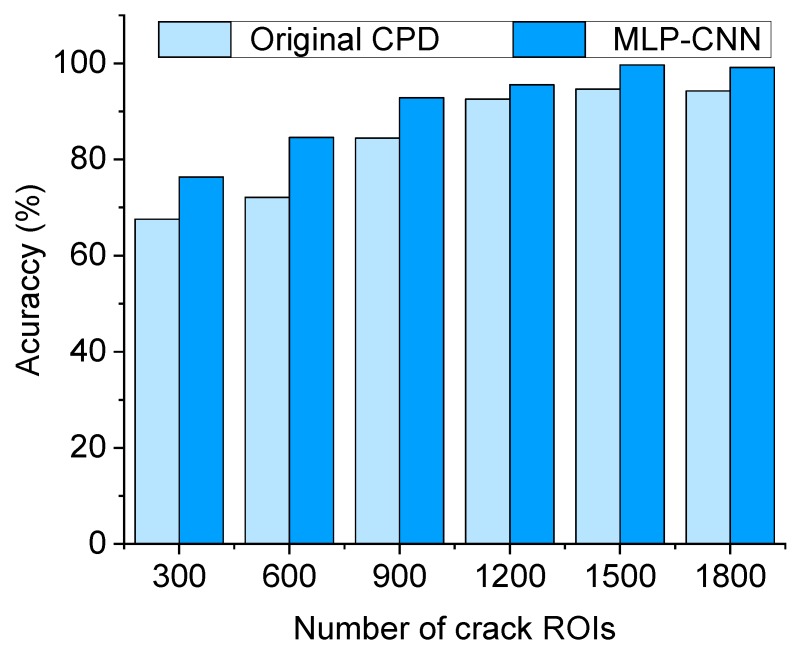
Accuracy of the original CPD and the MLP–CNN subject to different numbers of crack ROIs in the training set.

To further examine the crack detection accuracy, 5000 background ROIs combined with varied numbers of crack ROIs (from 300 to 1800) were used in the training set. As shown in Figure 18, the test accuracy of both the original CPD and the MLP–CNN tends to be stable after the number of crack ROIs exceeds 1500. It is notable that the accuracy enhancement with use of the MLP–CNN is especially significant under relatively small amounts of training data. 

**Figure 19:**
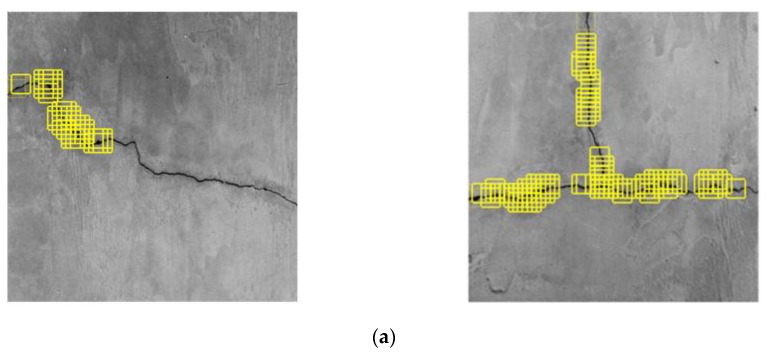

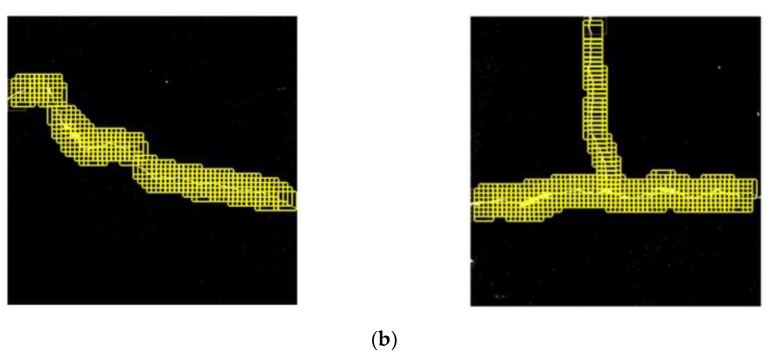
Crack position detection(CPD) effect based on (**a**) the original CPD and (**b**) MLP–CNN, under moderate level of noise influence.

Figure 19 shows the CPD results using a sliding window with the sliding step of 16 pixels. The results of the original CPD show large discontinuities along the main portion of the crack, whereas the MLP–CNN has an obviously superior accuracy of detection, by which all cracks are identified precisely, as shown in Figure 19b.

### 4.3. Crack Type Identification Subject to Moderate Noise Level

The accuracy of the CTI network changed from 99.3% to 98.8% with use of the MLP–CNN framework under moderate noise. Although there was a slight accuracy decrease of 0.6% after using MLP–CNN, additional analysis showed that the difference in accuracy before and after the MLP treatment was negligible. 

**Figure 20:**
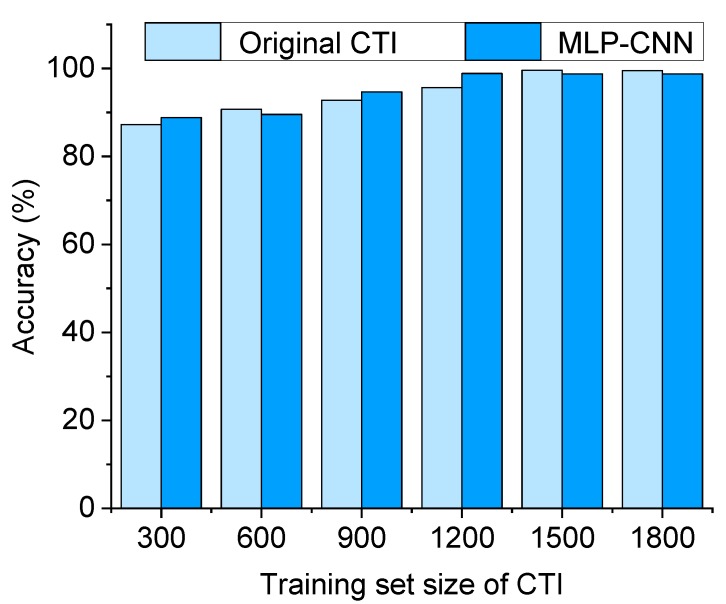
Accuracy of the original CTI and the MLP–CNN subject to different numbers of crack ROIs in the training set.

The variation of accuracy in crack type identification along with the increase of the size of the training set is shown in Figure 20. The abscissa represents the number of images (from 300 to 1800, with the increment of 300) in the training set that includes the five crack types. The ordinate shows the testing accuracy of crack type classification using the original CTI and the MLP–CNN, respectively. It can be seen that the overall accuracy of crack type identification using the original CTI and the MLP–CNN, respectively, under different sizes of training sets, is comparable. Moreover, the accuracy tends to remain stable along with the increase in size of the training set. To conclude, no obvious improvement of detection accuracy was obtained using MLP–CNN under a relatively low level of noise influence. Improvement in detection accuracy is expected to be achieved under higher noise levels. 

### 4.4. Crack Position Detection Subject to Severe Noise Influence

Severe noise influence subject to different sources, including light spots, blurs, and surface anomalies (e.g., uneven surface, low color contract, stains) is considered in this section, treated by the original CPD and the MLP–CNN framework.

#### 4.4.1. Light Spots

Light spots were simulated by first setting the center of the light source, i.e., (*x*_0_, *y*_0_). With *w* and *h* defined as the width and height of an image, *x*_0_ and *y*_0_ were set at *w*/3 and *h*/2, respectively. Thus, images including light spots were generated in accordance with:

(5) $$∆\left(x,y\right)=k\times \left(1-\frac{\sqrt{{\left(x-x_{0}\right)}^{2}+{\left(y-y_{0}\right)}^{2}}}{r}\right),$$

(6) L(x,y)=l(x,y)+∆(x,y).

where *k* is a parameter relating to the brightness of the light spot, which is equal to 0.4; *r* is the radius of the light source; ∆(x,y) is a matrix of the same size as the input image, representing the increase in the pixel values of the image. Adding it to the matrix of the original image l(x,y) gives L(x,y) after the lighting simulation.

**Figure 21:**
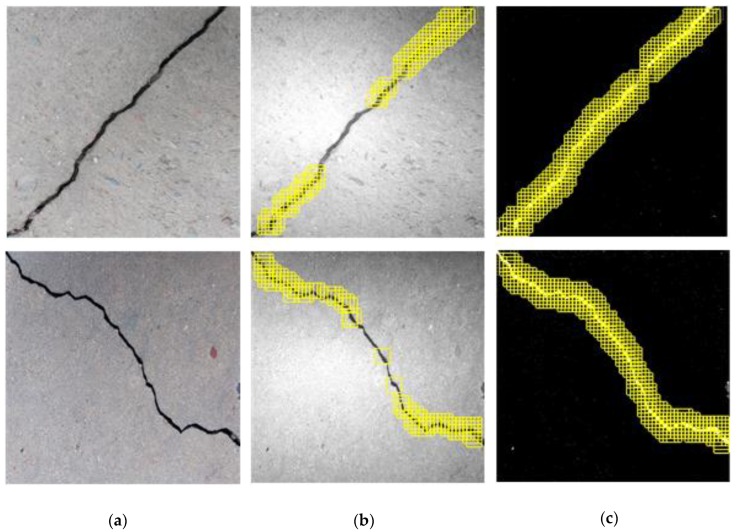
The (**a**) original crack images, and the results of crack position detection (CPD) based on (**b**) the original CPD and (**c**) the MLP–CNN, under the influence of light spots.

The closer pixel points were to the light source, the greater was their brightness value. 80 images after light spot treatment were randomly selected to form the test set. The crack position detection results obtained using the original CPD and the MLP–CNN are shown in Figure 21. It is seen that the original CPD fails to identify some main portions of the crack, which, interestingly, are located close to the light source. In contrast, the MLP–CNN shows significant resistance to light spot influence, leading to accurate identification results.

#### 4.4.2. Blurs

**Figure 22:**
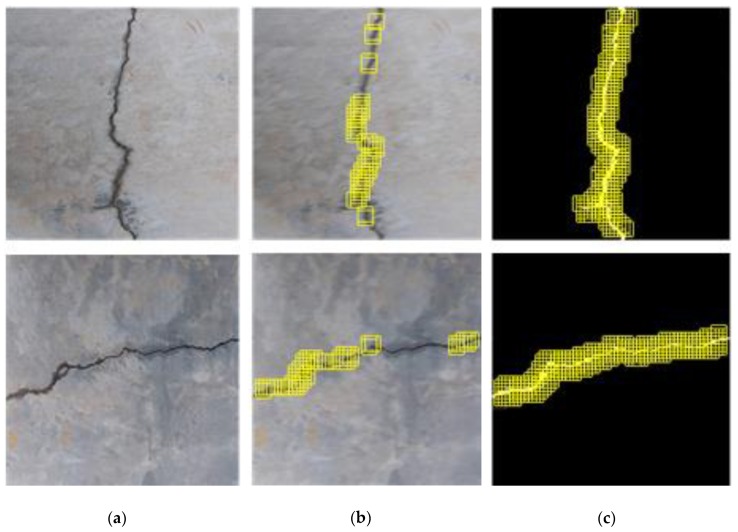
(**a**) Original crack images, and the results of crack position detection (CPD) based on (**b**) the original CPD and (**c**) the MLP–CNN, under the influence of blurs.

Motion blurs were used to simulate camera movement caused by environmental or human factors. The motion displacement was set to 20 pixels with motion angle of 15°. After the simulation of motion blur, 80 crack images were selected to form a blurred test set. The crack position detection results, under the blur noise, based on the original CPD and the MLP–CNN are shown in Figure 22. As is evident, the blurring of image results in poor crack recognition with use of the original CPD, causing serious discontinuity and even failure in crack identification, as seen from Figure 22b. Fortunately, the MLP–CNN largely increases the identification accuracy under the blurring conditions, so that all details of crack information are well maintained. 

#### 4.4.3. Surface Anomalies

**Figure 23:**
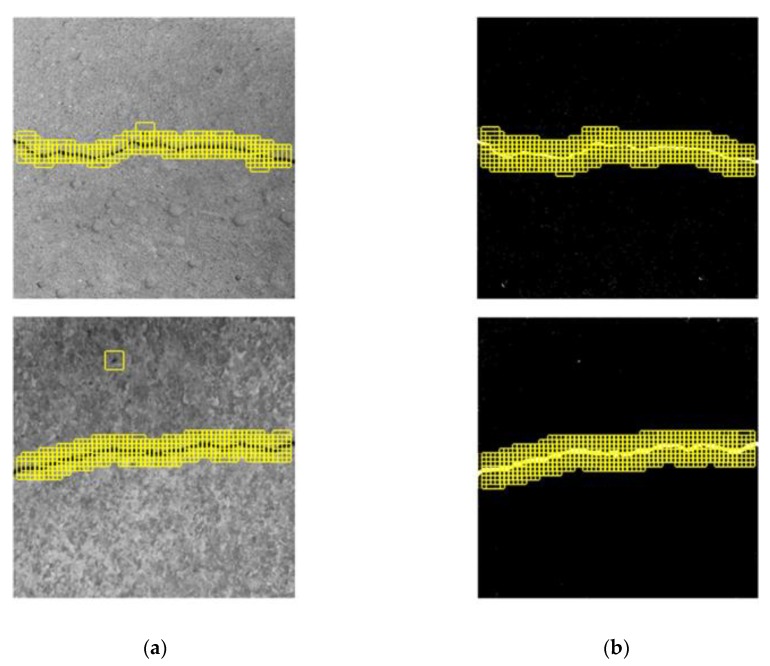
Results of crack position detection (CPD) based on (**a**) the original CPD and (**b**) the MLP–CNN, with uneven concrete surfaces.

**Figure 24:**
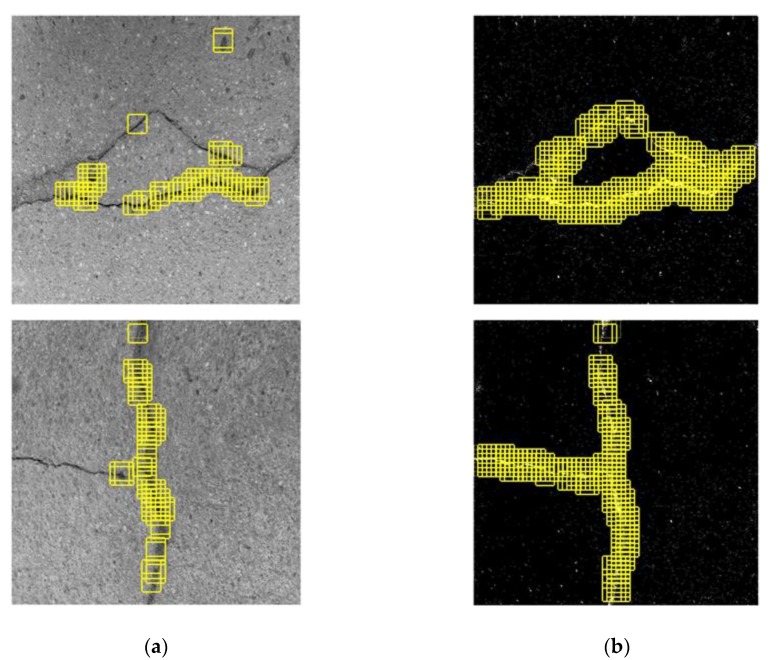
Results of crack position detection (CPD) based on (**a**) the original CPD and (**b**) the MLP–CNN, with low color contract between crack and background.

**Figure 25:**
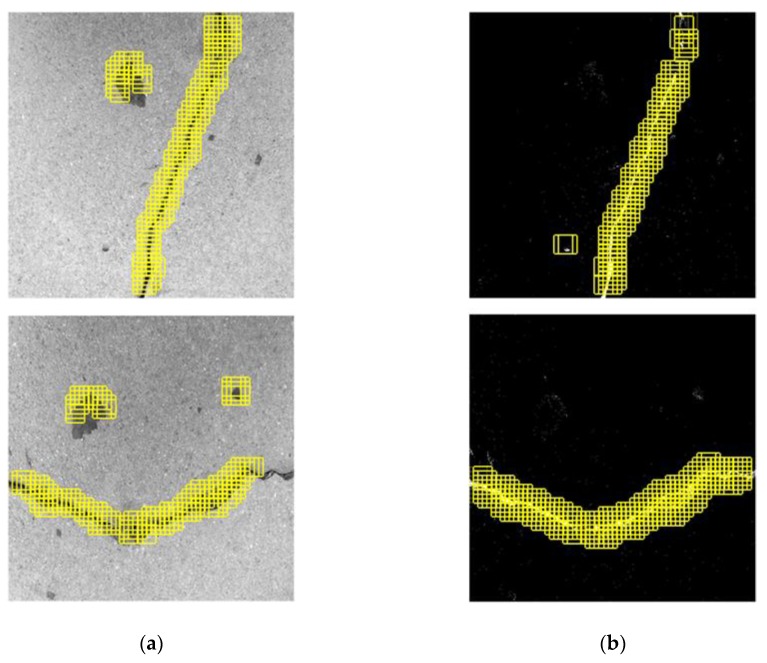
Results of crack position detection (CPD) based on (**a**) the original CPD and (**b**) the MLP–CNN, with stains in the background.

120 images subject to noise due to background surface anomalies in terms of wear, stains, etc., were selected to form the test set of surface anomalies. The detection results for uneven concrete surfaces are shown in Figure 23. It can be seen that the overall detection accuracy of the original CPD is satisfactory, but false alarms still exist in the intact region, as shown in Figure 23a. The false alarms can be effectively prevented with the use of MLP–CNN as shown in Figure 23b. Figure 24 shows the detection results subject to low contrast in color between crack and background, defined here as another type of surface anomaly. With the original CPD, several background ROIs were misclassified as crack ROIs, and some main portions of cracks were missed. In contrast, using MLP–CNN, the majority of crack information was well manifested, containing few misclassifications and rich crack information. Figure 25 shows the detection results with regard to surface stains, in which it can be seen that the stains further raise the issue of misclassification, with most stain areas in the background identified as cracks with use of the original CPD. Such issues are largely prevented with use of the MLP–CNN.

### 4.5. Crack Type Identification Subject to Severe Noise Influence

Table 4 shows four test sets used by the CTI network, each representing a specific type of noise, where moderate noise refers to noise influence as discussed in Section 4.2 and Section 4.3.

Table 5 presents the accuracy of the original CTI network and the MLP–CNN in crack type identification subject to different types and levels of noise influence. Apart from the observation under moderate noise influence, where the accuracy of the original CTI and the MLP–CNN is comparable, a clear difference in identification accuracy between the original CTI and the MLP–CNN can be seen under severe noise influence. Specifically, MLP–CNN improves the accuracy by 2.8% under light spot influence, 5.4% under blur influence, and 4.7% under surface anomaly influence. In summary, the stronger recognition accuracy of the MLP–CNN than that obtained using the CNN without the implementation of the MLP is demonstrated, particularly under the influence of severe noise.

## 5. Conclusions

In recognition of the influence of severe noise included in concrete surface images, which largely degraded the accuracy of existing methods in crack identification, a MLP–CNN framework was established in this paper relying on hybrid utilization of CNN and a multi-layered preprocessing technique, the key elements of which were homomorphic filtering and the Otsu thresholding method. With a binary nature and prominent crack features, the crack images processed by the MLP enabled a significant enhancement of accuracy and versatility for concrete crack identification based on CNN application.

Compared to the original CNN networks, i.e., CPD and CTI, the MLP–CNN framework proved able to improve crack identification. Specifically, under a moderate noise level, accuracy of CPD was increased by 3.1% using the MLP–CNN. However, the improvement in CTI could not be observed clearly. Severe noise influence was then introduced, with sources in terms of light spot, blur, or surface anomaly. Clear enhancement of crack identification was observed: (a) Subject to the noise influence of light spot and blur, a significant amount of information of large portions of cracks was misclassified with use of the original CPD, whereas the MLP–CNN identified the cracks precisely, with the majority of crack information preserved. For CTI, the MLP–CNN increased recognition accuracy by 2.8% and 5.4% under the influence of light spot and blur, respectively. (b) Subject to noise influence from surface anomalies, specifically uneven surface, low color contract between crack and background, and stains, the original CPD encountered problems of missing crack information as well as giving false alarms for the background region. These two drawbacks were well addressed by applying the MLP–CNN. For CTI, the MLP–CNN increased the recognition accuracy by 4.7%.

The MLP–CNN framework shows potential applications in accurate identification of surface cracks in concrete buildings such as bridges and dams. And its efficiency can be maximized in some future scenarios, for example, rapid capturing of massive concrete surface images using drones. The robustness and flexibility of the MLP–CNN framework can be further enhanced in future study by adjusting the CNN types and parameters and enlarging the datasets. More comprehensive information of cracks, such as depths and more patterns, is aimed to be recognized quantitatively. 

## Figures and Tables

**Table 1:** Ultimate accuracy and training time of ROI recognition by Alexnet, Lenet5, and CIFAR10, respectively.

CNN	Test accuracy (%)	Time
Alexnet	98.6	140 min 7 s
Lenet5	45.2	1 min 25 s
CIFAR10	94.6	8 min 12 s

**Table 2:** Specific structure of the CPD network (where *h*, *w*, and *d* are height, width, and depth, respectively; Conv is convolution layer; MX is MaxPooling layer; FC is fully connected layer; D is dropout layer; S is softmax layer; NOI is number of input; NOO is number of output).

**Layer**	**L1**	**L2**	**L3**	**L4**	**L5**	**L6**	**L7**	**L8**
Operator	Input	Conv	ReLU	MP	Conv	ReLU	MP	Conv
*h/w/d*	32/32/3	5/5/32	-	3/3/-	5/5/32	-	3/3/-	5/5/64
Stride	-	1	-	2	1	-	2	1
**L9**	**L10**	**Layer**	**L11**	**L12**	**L13**	**L14**	**L15**	**L16**
ReLU	MP	Operator	FC	ReLU	D	FC	S	Output
-	3/3/-	NOI	576	-	-	256	-	2
-	2	NOO	256	-	-	2	-	2

**Table 3:** Specific structure of the CTI network (BN is batch normalization layer; the meanings of the other abbreviations are consistent with those in Table 2).

**Layer**	**L1**	**L2**	**L3**	**L4**	**L5**	**L6**	**L7**	**L8**
Operator	Input	Conv	ReLU	BN	MP	Conv	ReLU	BN
*h/w/d*	227/227/3	11/11/96	-	-	3/3/-	5/5/128	-	-
Stride	-	4	-	-	2	1	-	-
**L9**	**L10**	**L11**	**L12**	**L13**	**L14**	**L15**	**L16**	**Layer**
MP	Conv	ReLU	Conv	ReLU	Conv	ReLU	MP	Operator
3/3/-	3/3/384	-	3/3/192	-	3/3/128	-	3/3/-	NOI
2	1	-	1	-	1	-	2	NOO
**L17**	**L18**	**L19**	**L20**	**L21**	**L22**	**L23**	**L24**	**L25**
FC	ReLU	D	FC	ReLU	D	FC	S	Output
9216	-	-	4096	-	-	4096	-	5
4096	-	-	4096	-	-	5	-	5

**Table 4:** Four test sets types and the number of pictures contained.

Test Set	Moderate Noise	Light Spot	Blur	Surface Anomaly
Size of each set	577	80	80	120

**Table 5:** Accuracy of crack type identification under different noise types and levels.

Noise Condition	Accuracy of CTI Network (%)	Accuracy of MLP–CNN (%)
Moderate noise	99.3	98.7
Light spot	86.5	89.3
Blur	82.6	88.0
Surface anomaly	89.6	94.3
